# Electron Scattering via Interface Optical Phonons with High Group Velocity in Wurtzite GaN-based Quantum Well Heterostructure

**DOI:** 10.1038/s41598-018-34441-4

**Published:** 2018-10-29

**Authors:** Kihoon Park, Ahmed Mohamed, Mitra Dutta, Michael A. Stroscio, Can Bayram

**Affiliations:** 1 Department of Electrical and Computer Engineering, University of Illinois at Urbana-Champaign, Urbana, Illinois 61801 USA; 2 Micro and Nanotechnology Laboratory, University of Illinois at Urbana-Champaign, Urbana, Illinois 61801 USA; 3 Department of Electrical and Computer Engineering, University of Illinois at Chicago, Chicago, Illinois 60607 USA

## Abstract

Here we present a detailed theoretical analysis of the interaction between electrons and optical phonons of interface and confined modes in a wurtzite AlN/GaN/AlN quantum well heterostructure based on the uniaxial dielectric continuum model. The formalism describing the interface and confined mode optical phonon dispersion relation, electron–phonon scattering rates, and average group velocity of emitted optical phonons are developed and numerically calculated. The dispersion relation of the interface phonons shows a convergence to the resonant phonon frequencies 577.8 and 832.3 cm^−1^ with a steep slope around the zone center indicating a large group velocity. At the onset of interface phonon emission, the average group velocity is small due to the large contribution of interface and confined mode phonons with close-to-zero group velocity, but eventually increases up to larger values than the bulk GaN acoustic phonon velocity along the wurtzite crystal *c*-axis (8 nm/ps). By adjusting the GaN thickness in the double heterostructure, the average group velocity can be engineered to become larger than the velocity of acoustic phonons at a specific electron energy. This suggests that the high group velocity interface mode optical phonons can be exploited to remove heat more effectively and reduce junction temperatures in GaN-based heterostructures.

## Introduction

GaN-based semiconductors are of great interest in the electronics and optoelectronics communities because they possess large electronic bandgaps (3.4 eV) suitable for fabricating semiconductor lasers with wavelengths in blue and ultraviolet^1^ as well as electronic devices designed to tolerate high electric fields (3.3 MV/cm) and elevated operating temperatures (700 °C)^2^. In particular, AlGaN/GaN high electron mobility transistors (HEMTs) are among the most promising devices for high-power applications^3^. The spontaneous and piezoelectric polarization fields of this heterostructure allow the GaN layer to form a high-density electron channel through which electrons can flow with high saturation velocity (2.5 × 10^7^ cm/s); this is partly due to the optical phonons with high energy (*ħω*_LO_ = 92 meV) in GaN^4^. The electron velocity saturation occurs with the onset of emission of these optical phonons and therefore their energy roughly determines the electron saturation velocity according to *v*_0_ ≈ [*ħω*_LO_/*m*]^1/2^, where *m* is the effective electron mass. Taking a closer look into the material, the large mismatch between the cation and anion masses causes a large splitting between the energies of the optical and acoustic phonon branches which raises the energy of optical phonons^5^. The drawback associated with these high energy optical phonons is their short interaction time with electrons compared to the long decay time into acoustic phonons. As in common semiconductors, heat in GaN is carried mainly by the long wavelength acoustic phonons^6^. To remove the excessive heat that is generated in the electron channel by the collision of hot electrons and the lattice (at the rate of ~10 THz)^7^, the non-equilibrium optical phonons (whose propagation velocity is close to zero) must decay into acoustic phonons. The process is known as the Ridley process^8^ and the decay time is reported to be ~5 ps which is much longer than the electron interaction time of ~9 fs^9^. This mismatch between the optical phonon generation rate and the relaxation rate into acoustic phonons results in a large accumulation of optical phonons in a localized region at the channel and eventually causes the electronic properties to degrade^10,11^.

In this work, we propose a novel phonon engineering technique that portends applications related to reducing the maximum temperature of the localized hotspot in GaN-based devices. To enhance the heat dissipation efficiency, a double heterostructure consisting of AlN/GaN/AlN is introduced to exploit properties of the interface mode optical phonons. Unlike bulk optical phonons, the mixing between available phonon frequencies of AlN and GaN induces an optical phonon mode that possesses a high propagation velocity at the heterointerface. The existence of these optical phonon modes has been corroborated by Raman studies on AlN/GaN superlattices^12,13^. The introduction of these interface mode phonons provides an extra channel through which heat can be removed. Here, we employ the uniaxial dielectric continuum model to theoretically examine the properties of interface and confined mode optical phonons and their interaction with hot electrons.

## Optical Phonon Mode Dispersion In Wurtzite Crystals

In the double heterostructure of interest in this work, which is a GaN quantum well sandwiched by two AlN layers (AlN/GaN/AlN), there exists four distinct classes of optical phonon modes: the interface, confined, half-space, and propagating modes^14^. Among these four optical phonon modes, the electrons that are confined in the GaN quantum well mostly interact with the interface and confined phonons; the effect of the half-space and propagating modes on the electrons is negligible in this system^15^. Here, therefore, we only consider the electron scattering with interface and confined mode optical phonons.

In a heterostructure configuration, the available optical phonon modes and the phonon frequencies for each mode are determined by the relation between the dielectric constant functions. The frequency-dependent dielectric functions parallel *ε*_z_ and perpendicular *ε*_t_ to the *z*-axis are given as^16^:

1 $${\varepsilon }_{{\rm{z}}}(\omega )={\varepsilon }_{{\rm{z}}}^{\infty }\frac{{\omega }^{2}-{\omega }_{{\rm{Lz}}}^{2}}{{\omega }^{2}-{\omega }_{{\rm{z}}}^{2}}$$

2 $${\varepsilon }_{{\rm{t}}}(\omega )={\varepsilon }_{{\rm{t}}}^{\infty }\frac{{\omega }^{2}-{\omega }_{{\rm{Lt}}}^{2}}{{\omega }^{2}-{\omega }_{{\rm{t}}}^{2}}$$

where *ω* is the phonon frequency, *ω*_Lz_, *ω*_z_, *ω*_Lt_, and *ω*_t_ are the characteristic frequencies of A_1_(LO: longitudinal-optical), A_1_(TO: transverse-optical), E_1_(LO), and E_1_(TO) optical phonon modes, respectively. For the AlN/GaN/AlN quantum well, two sets of material parameters are required such that we obtain four dielectric functions, namely *ε*_1z_, *ε*_1t_, *ε*_2z_, and *ε*_2t_, where the subscripts 1 and 2 indicate the GaN and AlN, respectively. With *ω* = 0, the Lyddane-Sachs-Teller relation is recovered and the static dielectric constants are obtained. Throughout this paper, we take the *z*-axis along the *c*-axis of the wurtzite crystal [0001] and perpendicular to the heterointerfaces.

The phonon frequencies and dielectric constants for bulk GaN and AlN used in the calculations are listed in Table 1^17,18^. Using these frequency-dependent dielectric functions of bulk GaN and AlN, the phonon frequencies and available phonon modes of the AlN/GaN/AlN quantum well are deduced by the dielectric continuum model. Notice that the phonon frequencies are listed in units of cm^−1^. In the following calculations, whenever appropriate, they are converted into units of s^−1^. We assume that the two high-frequency dielectric constants are identical, i.e., ${\varepsilon }_{{\rm{z}}}^{\infty }={\varepsilon }_{{\rm{t}}}^{\infty }={\varepsilon }^{\infty }$^19^.

**Table 1:** Material constants used in the numerical calculations.

Material Constants	Symbols	GaN	AlN
A_1_(TO) phonon frequency^a^	*ω*_z_ (cm^−1^)	531	611
E_1_(TO) phonon frequency^a^	*ω*_t_ (cm^−1^)	559	671
A_1_(LO) phonon frequency^a^	*ω*_Lz_ (cm^−1^)	734	890
E_1_(LO) phonon frequency^a^	*ω*_Lt_ (cm^−1^)	741	912
High-frequency dielectric constant^b^	*ε* ^∞^	5.35	4.77

^a^Phonon frequencies taken from ref.^17^.

^b^Dielectric constants taken from ref.^18^.

The conditions imposed on the available interface mode optical phonon frequency are

3 $${\varepsilon }_{1z}{\varepsilon }_{1{\rm{t}}} > 0,\,{\varepsilon }_{2z}{\varepsilon }_{2{\rm{t}}} > 0,\,{\rm{and}}\,{\varepsilon }_{1z}{\varepsilon }_{2{\rm{z}}} < 0.$$

For confined modes, the conditions are

4 $${\varepsilon }_{1z}{\varepsilon }_{{\rm{1t}}} < 0\,{\rm{and}}\,{\varepsilon }_{2z}{\varepsilon }_{{\rm{2t}}} > 0.$$

**Figure 1:**
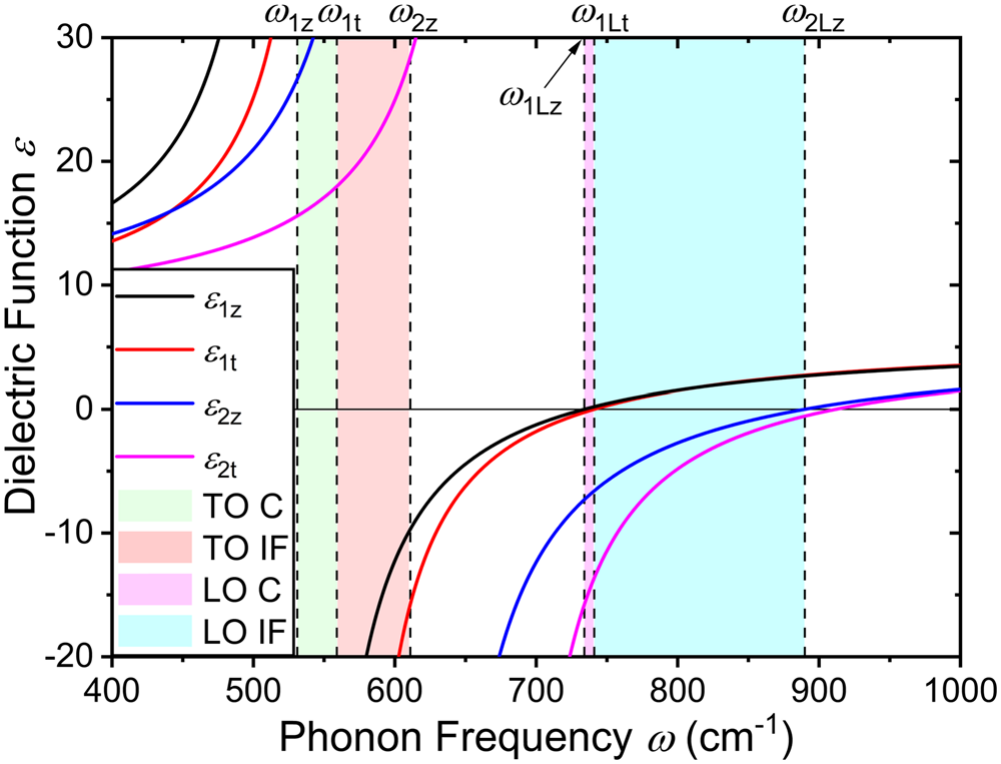
The dielectric constants as a function of phonon frequency are plotted. The characteristic frequencies of the dielectric functions which define the range of available interface (IF) and confined (C) mode phonon frequencies are indicated by vertical dashed lines. The region shaded in red (*ω*_1t_, *ω*_2z_) is where the interface phonons associated with the TO phonon modes of bulk GaN and AlN are defined. The region shaded in blue (*ω*_1Lt,_ *ω*_2Lz_) is where the interface phonons associated with the LO phonon modes of bulk GaN and AlN are defined. The regions shaded in green (*ω*_1z_, *ω*_1t_) and magenta (*ω*_1Lz_, *ω*_1Lt_) are where the confined phonons are defined.

To clearly illustrate the available range of phonon frequencies for each mode, the four dielectric constants as a function of phonon frequency are shown in Fig. 1. The characteristic frequencies of the dielectric functions which define the phonon frequency ranges are indicated by vertical dashed lines. According to the conditions in Eq. (3), the interface phonons are allowed in two phonon frequency intervals, (*ω*_1t_, *ω*_2z_) and (*ω*_1Lt_, *ω*_2Lz_). Since the former (latter) interval corresponds to the TO (LO) phonon frequencies of GaN and AlN, we label the phonon modes that lie in this frequency range as TO (LO) interface phonons. These intervals are indicated in the figure as red and blue shaded regions, respectively. Similarly, according to the conditions in Eq. (4), the confined phonons are allowed in two phonon frequency intervals, (*ω*_1z_, *ω*_1t_) and (*ω*_1Lz_, *ω*_1Lt_). The characteristic phonon frequencies associated with these intervals are from the TO and LO phonon frequencies of GaN, and hence we label them as TO confined and LO confined phonons. The TO and LO confined phonon frequency ranges are shown in the figure as green and magenta shaded regions, respectively.

### Interface modes

The dispersion relations for the symmetric ${q}_{{\rm{S}}}^{{\rm{IF}}}$ and asymmetric ${q}_{{\rm{A}}}^{{\rm{IF}}}$ interface phonon modes are described by^20^

5 $${q}_{{\rm{S}}}^{{\rm{IF}}}=\frac{1}{2\alpha d}\,\mathrm{ln}[\frac{{\xi }_{1}+{\xi }_{2}}{{\xi }_{1}-{\xi }_{2}}]$$

6 $${q}_{{\rm{A}}}^{{\rm{IF}}}=\frac{1}{2\alpha d}\,\mathrm{ln}[\frac{{\xi }_{1}+{\xi }_{2}}{{\xi }_{2}-{\xi }_{1}}]$$

where $\alpha =\frac{1}{2}\sqrt{|{\varepsilon }_{{\rm{1t}}}(\omega )/{\varepsilon }_{{\rm{1z}}}(\omega )|}$, ${\xi }_{1}=\sqrt{|{\varepsilon }_{{\rm{1z}}}(\omega ){\varepsilon }_{{\rm{1t}}}(\omega )|}$, ${\xi }_{2}=\sqrt{|{\varepsilon }_{{\rm{2z}}}(\omega ){\varepsilon }_{{\rm{2t}}}(\omega )|}$, and *d* is the quantum well thickness. The GaN quantum well thickness *d* is set to a default value of 5 nm in the following calculations unless otherwise specified. The phonon wave vectors ${q}_{{\rm{S}}}^{{\rm{IF}}}$ and ${q}_{{\rm{A}}}^{{\rm{IF}}}$ must be real and positive which implies that symmetric and asymmetric modes are distinguished based on the polarity of *ξ*_1_ – *ξ*_2_.

The resonant interface phonon frequency is obtained from *ξ*_1_ = *ξ*_2_. The *ω*_TO,res_ and *ω*_LO,res_ are the TO and LO resonant interface frequencies where *ξ*_1_ and *ξ*_2_ are equal in the TO and LO phonon frequency range, respectively. These frequencies are calculated as *ω*_TO,res_ = 577.8 cm^−1^ and *ω*_LO,res_ = 832.3 cm^−1^. From the definition of the symmetric and asymmetric phonon wave vectors, the symmetric mode is only defined in the frequency range where *ξ*_1_ > *ξ*_2_ and the asymmetric mode is only defined in the range where *ξ*_1_ < *ξ*_2_. Combined with the constraints of the dielectric constants, the symmetric TO interface modes can only be defined in the phonon frequency range (*ω*_1t_, *ω*_TO,res_) and the symmetric LO modes in the range (*ω*_LO,res_, *ω*_2Lz_). Similarly, the asymmetric TO interface phonons are only defined in (*ω*_TO,res_, *ω*_2z_) and the asymmetric LO modes in (*ω*_1Lt_, *ω*_LO,res_).

### Confined modes

The dispersion relations for the symmetric ${q}_{{\rm{S}}}^{C}$ and asymmetric ${q}_{{\rm{A}}}^{C}$ confined phonon modes are described by^20^

7 $$\begin{array}{rcl}{q}_{{\rm{S}}}^{{\rm{C}}} & = & \frac{1}{\alpha d}[n\pi +\mu \,\arctan \frac{{\xi }_{2}}{{\xi }_{1}}]\\ n & = & 1,2,3\ldots \,{\rm{and}}\,0\,{\rm{if}}\,\mu =1\end{array}$$

8 $$\begin{array}{rcl}{q}_{{\rm{A}}}^{{\rm{C}}} & = & \frac{1}{\alpha d}[n\pi -\mu \,\arctan \frac{{\xi }_{1}}{{\xi }_{2}}]\\ n & = & 1,2,3\ldots \,{\rm{and}}\,0\,{\rm{if}}\,\mu =\mbox{--}1\end{array}$$

where *μ* = sign[*ε*_1z_(*ω*)*ε*_2z_(*ω*)] and *n* is the quantum number for symmetric and asymmetric confined modes.

**Figure 2:**
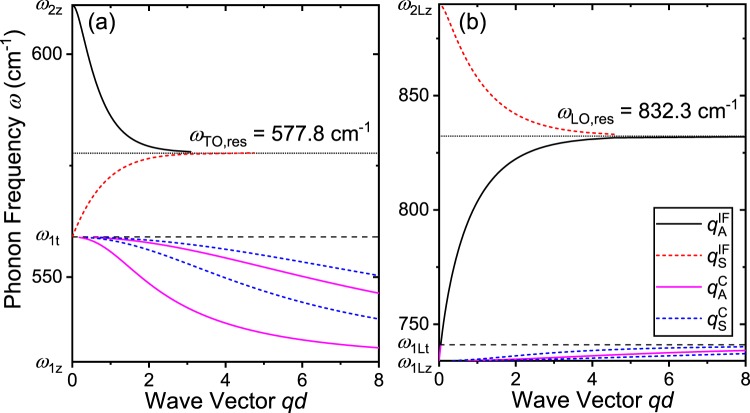
Dispersion relation of asymmetric interface (${q}_{{\rm{A}}}^{{\rm{IF}}}$), symmetric interface (${q}_{{\rm{S}}}^{{\rm{IF}}}$), asymmetric confined (${q}_{{\rm{A}}}^{{\rm{C}}}$), and symmetric confined (${q}_{{\rm{S}}}^{{\rm{C}}}$) mode phonons of the AlN/GaN/AlN quantum well in the (**a**) low frequency and (**b**) high frequency region are plotted. Only a few of the confined mode phonons (two modes each for the asymmetric and symmetric branch with lowest quantum numbers) are plotted. Phonon modes associated with TO phonon modes of AlN and GaN are shown in the (**a**) lower frequency region whereas those associated with LO phonon modes are in the (**b**) higher frequency region. The interface phonon resonant frequencies are shown in dotted horizontal lines and the other characteristic frequencies are shown in dashed horizontal lines. The *x*-axis is shown in the dimensionless wave vector *qd*.

The interface and confined mode phonon dispersion relations [Eqs (5)–(8)] are shown in Fig. 2. The subscripts *A* and *S* indicate asymmetric and symmetric modes, and superscripts *IF* and *C* indicate interface and confined modes, respectively. The low frequency modes that are associated with the TO phonon frequencies of GaN and AlN are plotted in (a) and the high frequency modes that are associated with the LO phonon frequencies are plotted in (b). The symmetric modes are shown in dashed lines and asymmetric modes are shown in solid lines for both interface and confined phonons. The characteristic phonon frequencies, which separate the interface modes from the confined modes, and resonant interface phonon frequencies are indicated with horizontal lines. In terms of phonon energy, the LO interface phonon modes $(91.9 < \hslash {\omega }_{{\rm{LO}}}^{{\rm{IF}}} < 110.3\,{\rm{eV}})$ are higher in energy compared to the TO phonon modes ($69.3 < \hslash {\omega }_{{\rm{TO}}}^{{\rm{IF}}} < 75.8\,{\rm{eV}}$). The TO and LO resonant interface phonon energies are 71.7 and 103.2 meV, respectively. Compared to interface phonon modes, more than a pair of symmetric and asymmetric confined modes exist in each phonon frequency interval. Here, we only plot two of each symmetric and asymmetric confined modes that mostly contribute to the electron–phonon scattering process. However, in principle, there are an infinite amount of modes available. It should be noted that for the confined modes, the dispersion curves asymptotically approach a characteristic phonon frequency of GaN (i.e., *ω*_1z_ for TO confined modes and *ω*_1Lt_ for LO confined modes), whereas for the interface modes the curves approach the resonant frequency with increasing wave vector. This interface phonon dispersion relation gives rise to a phonon emission threshold energy in the electron–phonon scattering process that does not correspond to an energy of the characteristic phonons of either AlN or GaN.

Another important feature in the dispersion relation is that these interface phonon modes have a nonzero slope at the zone center. This indicates that the group velocity *v*_*g*_ = *dω*/*dq* is nonzero and that optical phonons generated at the interface will not stay where they were generated (as bulk optical phonons do) but will propagate along the interface. Notice that the *x*-axis is set to the dimensionless product of phonon wave vector *q* and the quantum well thickness *d*. Therefore, the group velocity will increase with increasing quantum well thickness due to the stronger dispersion.

## Electron–Phonon Scattering Rate

To investigate the electron scattering rate with confined and interface mode phonons, we adapt the expression from ref.^15^:

9 $$\frac{1}{{\tau }^{{(}_{e}^{a})}}=\pm \,\frac{2m{e}_{0}^{2}}{{\hslash }^{2}}\sum _{n}{\int }_{{\omega }_{1}}^{{\omega }_{2}}\frac{\{N(\omega )+\frac{1}{2}\mp \frac{1}{2}\}D(q,\omega )\sigma [\{\frac{\omega }{{q}^{2}}\pm \frac{\hslash }{2m}\}\frac{dq}{d\omega }-\frac{1}{q}]}{[\frac{q}{2}\pm \frac{m}{\hslash }\{\frac{\omega }{q}-\frac{d\omega }{dq}\}]\sqrt{\frac{1}{m}\chi ({\bf{k}},q,\omega )}}d\omega $$

with

10 $$\chi ({\bf{k}},q,\omega )=\frac{{\hslash }^{2}{{\bf{k}}}^{2}}{m}-\frac{{\hslash }^{2}{q}^{2}}{4m}\pm \hslash \omega -m\frac{d\omega }{dq}$$

11 $$\sigma =\{\begin{array}{ll}0 & {\rm{for}}\,\chi ({\bf{k}},q,\omega ) < 0\\ 1 & {\rm{otherwise}}\end{array}$$

where *m* = m^*^m_0_, the dimensionless effective mass^21^ is m^*^ = 0.22, *m*_0_ is the electron rest mass, *e*_0_ is the elementary charge, and $N(\omega )=\frac{1}{{e}^{\hslash \omega /{k}_{{\rm{B}}}T}-1}$ is the phonon occupation number. The upper (lower) signs are taken when considering electron absorption (emission) scattering process. The summation over the quantum number *n* [identical to the ones in Eqs (7) and (8)] is included to consider scattering with all possible symmetric and asymmetric confined mode phonons. For each *n*, the proper dispersion relation between *q* and *ω* should be imposed through Eqs (7) or (8). In our numerical calculation of the scattering rates, we find that a practical number for the upper limit is *n* = 5; contributions to the electron–phonon scattering rate from confined modes with *n* larger than 5 are negligible. Also, the symmetric and asymmetric confined modes must be considered separately as the dispersion relations are different from each other. For the case of interface modes, the summation is omitted because only one interface mode exists in a given range of phonon frequency [*ω*_1_, *ω*_2_].

In the original formula^15^, where the integral is assessed over the angle *θ* between the phonon wave vector *q* and the optical axis *c*, the lower and upper limits of the integral are set to *θ* = 0 and 2π. In order to separately calculate the matrix elements of the Fermi golden rule for each phonon mode of uniaxial wurtzite crystals, the formula with the integral over *θ* is transformed into Eq. (9) where the integral is over *ω*. Considering the energy and momentum conservation of the electron–phonon scattering process, the limits of the integral over *ω* may also be transformed according to the relations:

12 $$\cos \,\theta =\{\begin{array}{ll}\frac{m\omega }{kq\hslash }-\frac{q}{2k} & {\rm{for}}\,{\rm{absorption}}\\ \frac{m\omega }{kq\hslash }+\frac{q}{2k} & {\rm{for}}\,{\rm{emission}}\end{array}$$

However, noticing that *q* is also a complex function of *ω* with different dispersion relations for different phonon modes [Eqs (5)–(8)] and that the integral must be calculated for different electron energies ${E}_{{\bf{k}}}={\hslash }^{2}{{\bf{k}}}^{2}/2m$, it is impractical to solve the nonlinear equation. Alternatively, we include a conditional variable *σ* to take care of the relation Eq. (12). Since the implications of the argument inside the square root of the denominator of Eq. (9) [*χ*(**k**, *q*, *ω*)/*m*] is identical to Eq. (12), we can evaluate the expression *χ*(**k**, *q*, *ω*) and keep the square root as a real value by imposing Eq. (11). This way, we can simply set the integral limits to the phonon frequencies where the phonon mode of interest is defined. For example, the upper and lower limits are set to *ω*_1_ = *ω*_1Lt_ and *ω*_2_ = *ω*_2Lz_, respectively, for the case of LO interface phonons.

For the function *D*(*q*, *ω*), depending on the phonon mode of interest it may be expressed as

13 $${D}^{{\rm{IF}}}=\frac{{{\rm{Y}}}^{4}{[\frac{{\cos }^{2}({k}_{1}\frac{d}{2})\cosh (\alpha qd)}{{k}_{2}+\beta q}+\frac{\{2{\cos }^{2}({k}_{1}\frac{d}{2}){\alpha }^{2}{q}^{2}+{k}_{1}^{2}\}\sinh (\alpha qd)+\alpha q{k}_{1}\cosh (\alpha qd)\sin ({k}_{1}d)}{2\alpha q({\alpha }^{2}{q}^{2}+{k}_{1}^{2})}]}^{2}}{{\cosh }^{2}(\alpha qd)\zeta {(\beta )}^{+}+\alpha qd\zeta {(\alpha )}^{-}+\,\sinh (\alpha qd)\zeta {(\alpha )}^{+}}$$

or

14 $${D}^{{\rm{C}}}=\frac{{{\rm{Y}}}^{4}{[\frac{{\cos }^{2}({k}_{1}\frac{d}{2})\cos (\alpha qd)}{{k}_{2}+\beta q}+\frac{\{2{\cos }^{2}({k}_{1}\frac{d}{2}){\alpha }^{2}{q}^{2}-{k}_{1}^{2}\}\sin (\alpha qd)-\alpha q{k}_{1}\cos (\alpha qd)\sin ({k}_{1}d)}{2\alpha q({\alpha }^{2}{q}^{2}-{k}_{1}^{2})}]}^{2}}{{\cos }^{2}(\alpha qd)\zeta {(\beta )}^{+}+\alpha qd\zeta {(\alpha )}^{+}+\,\sin (\alpha qd)\cos (\alpha qd)\zeta {(\alpha )}^{-}}$$

with

15 $${\rm{Y}}={[\frac{\sin ({k}_{1}d)}{2{k}_{1}}+\frac{d}{2}+\frac{{\cos }^{2}({k}_{1}d/2)}{{k}_{2}}]}^{-\frac{1}{2}}$$

where *ζ*^±^(*α*) = (1/2*α*)(∂*ε*_1t_/∂*ω*) ± 2*α*(∂*ε*_1z_/∂*ω*), *ζ*^±^(*β*) = (1/2*β*)(∂*ε*_2t_/∂*ω*) ± 2*β*(∂*ε*_2z_/∂*ω*), $\beta =\frac{1}{2}\sqrt{|{\varepsilon }_{2t}(\omega )/{\varepsilon }_{2z}(\omega )|}$, and *k*_1_ and *k*_2_ are the magnitudes of the electron wave vector inside and outside of the typical finite quantum well. Only the electronic ground state of the quantum well is considered and a conduction band offset of 2.3 eV between AlN and GaN is assumed^22^.

## Group Velocity Of Emitted Optical Phonons

**Figure 3:**
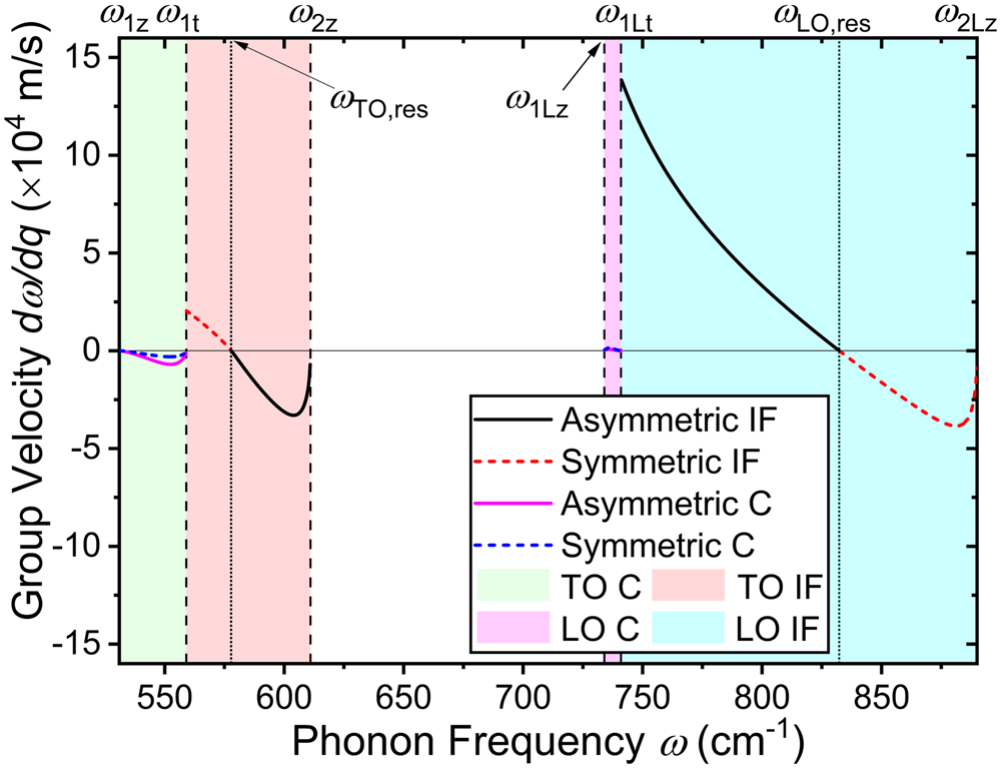
The numerically calculated group velocities of interface and confined mode phonons are shown as a function of phonon frequency. For both interface and confined modes, the solid and dashed lines indicate the asymmetric and symmetric phonon modes, respectively. The group velocity of interface phonons go to zero close to the TO and LO resonant frequency (*ω*_TO,res_ and *ω*_LO,res_ shown in vertical dotted lines). Maximum group velocity of 138 km/s occurs at *ω* = *ω*_1Lt_ for the interface mode phonons. This value is almost 20 times larger than the maximum group velocity of the confined mode phonons.

In Eq. (9), the phase (*v*_*p*_ = *ω*/*q*) and group velocity (*v*_*g*_ = *dω*/*dq*) of the phonon modes (or its reciprocal) are frequently used in the equation. Given the complex form of the phonon wave vector *q*, instead of expressing these velocities in a closed form equation, we calculate the velocities numerically. The group velocity is not only a component of the integral but also a crucial factor that describes the behavior of the phonons, and furthermore, the thermal characteristics of the system. Figure 3 shows the group velocity of the interface and confined phonon modes as a function of phonon frequency. As expected by the dispersion relation shown in Fig. 2, the interface mode phonons possess a considerably larger group velocity compared to the confined mode phonons. The largest confined mode group velocity is less than 7 km/s at ω ~ 552 cm^−1^ (TO mode), whereas the largest interface mode group velocity reaches up to 138 km/s (or 138 nm/ps) at *ω* = *ω*_1Lt_ (LO mode). It is easily deduced that if the full spectrum of interface phonon modes can be utilized, the phonons can carry a portion of the generated heat away along the heterointerface. This would be an additional heat transport mechanism, on top of the always existing acoustic phonon heat transport, that can help the system dissipate heat more efficiently.

**Figure 4:**
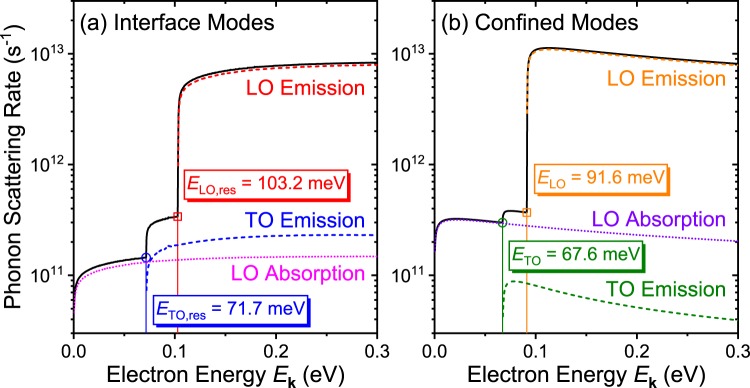
(**a**) Interface and (**b**) confined mode phonon scattering rates are calculated and plotted as a function of electron energy. For both modes, the total interface phonon scattering rate combining all phonon modes (including the TO absorption scattering rate) is shown as the black solid line. The droplines with symbols are shown to indicate the threshold energies of TO emission and LO emission scattering. For interface mode scattering, these energies correspond to the TO and LO interface phonon resonant frequency energies *ħω*_TO,res_ = 71.7 meV and *ħω*_LO,res_ = 103.2 meV, respectively. For confined mode scattering, the threshold energies are at the vicinity (but not a complete match) of the TO and LO phonon energies of GaN, *ħω*_1z_ = 65.8 meV and *ħω*_1Lt_ = 91.9 meV, respectively.

To better understand the electron interaction with these interface and confined mode phonons, we evaluate Eq. (9) and calculate the scattering rates between the electrons and optical phonons. Figure 4 shows the (a) interface and (b) confined mode phonon scattering rates as a function of electron energy *E*_**k**_. For both (a) interface and (b) confined modes, the black solid line shows the total scattering rate which combines all contributions from each process indicated as dashed and dotted color lines. The general behavior of the scattering rate curves for both modes are similar. The LO emission scattering rates [(a) red and (b) orange dashed lines] start to dominate once the electron energy exceeds the threshold energy. The TO absorption processes are negligible compared to the others (not plotted in the figures). Due to mode mixing in wurtzites, the TO emission scattering rate is comparable to the LO absorption scattering rate. This causes the total scattering rate to show a two-step-like shape. The total scattering rate of both modes show roughly similar values that converge to ~10^13^ s^−1^ with *E*_**k**_ = 0.3 eV in the current system where the GaN thickness is set to *d* = 5 nm. As shown in Fig. 3, since we know that the interface mode phonons typically show larger group velocity, a comparable scattering rate between the interface and confined mode phonons indicates that the average phonon velocity of the phonons emitted due to these processes may be large enough to help dissipate the heat.

Focusing on the interface mode LO emission scattering rate [red dashed curve in (a)], we observe that the emission threshold energy is 103.2 meV. This energy corresponds to the energy of the LO resonant phonon frequency (*ω*_LO,res_ = 832.3 cm^−1^) shown as the horizontal dotted line in Fig. 2(b). The LO phonon emission process through electron–phonon scattering can only occur when the electron has, at least, more energy than the phonon to be emitted; of course, more specifically, the condition described by Eq. (12) must be satisfied. The emission threshold energy of 103.2 meV indicates that phonons with at least this amount of energy are most likely to be emitted. From Fig. 3, we see that the group velocity of phonons with the resonant frequencies *ω*_LO,res_ and *ω*_TO,res_ are zero. The TO emission threshold energy (71.7 meV) also corresponds to the energy of TO resonant phonon frequency (*ω*_TO,res_ = 577.8 cm^−1^). These observations of Fig. 4 suggest that, however large the group velocity of interface phonons *could* be, simply increasing the interface phonon mode scattering rate with respect to the confined phonon mode scattering rate may not improve the heat dissipation efficiency of the system.

For the confined modes in Fig. 4(b), the TO and LO emission threshold energies are shown as 67.6 and 91.6 meV, respectively. The TO emission threshold is slightly larger than *ħω*_1z_ = 65.8 meV. As shown in the dispersion curve in Fig. 2(a), the confined mode phonon frequencies asymptotically approach *ω*_1z_ with increasing phonon wave vector *q*. As the electron energy *E*_**k**_ increases, the energy and momentum conservation condition Eq. (12) [or Eq. (10) >0] is first satisfied at a considerably large *q*. It is revealed from the scattering rate calculation that this *q* is obtained when the phonon frequency *ω* is close to *ω*_1z_ = 531 cm^−1^, but slightly larger. Similarly, the LO emission threshold is slightly smaller than *ħω*_1Lt_ = 91.9 meV and this is due to the dispersion, shown in Fig. 2(b), approaching *ω*_1Lt_ with increasing *q*. Since Fig. 2 is shown with the dimensionless *x*-axis wave vector *qd*, it is apparent that the emission threshold energies will also be affected by the GaN thickness *d*.

**Figure 5:**
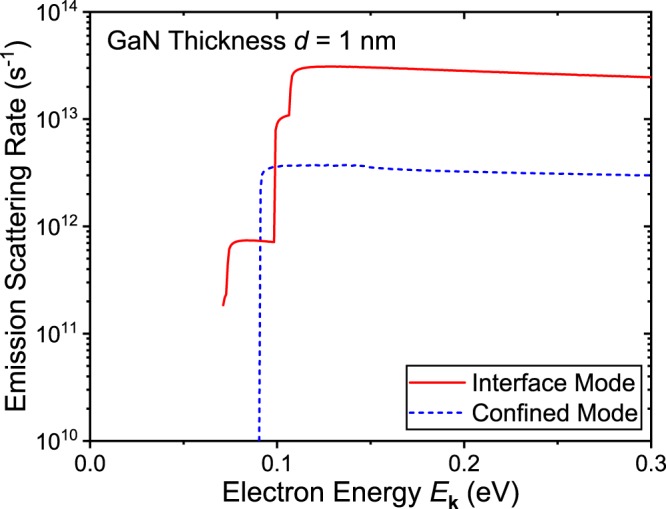
Interface and confined mode phonon emission scattering rates of a AlN/GaN/AlN double heterostructure with GaN thickness of *d* = 1 nm is plotted. Compared to the *d* = 5 nm case, the interface mode scattering rate (red solid line) show a factor of 3 increase, whereas the confined mode scattering rate (blue dashed line) show a factor of 2.5 decrease. Overall, the interface phonon scattering rate is approximately 8 times larger than the confined mode scattering rate with GaN thickness of *d* = 1 nm.

Figure 5 shows the interface and confined mode phonon emission scattering rates of an AlN/GaN/AlN double heterostructure with GaN thickness of *d* = 1 nm. Compared to the previous *d* = 5 nm case shown in Fig. 4, indeed, the interface mode scattering rate (red solid line) becomes approximately 8 times larger than the confined mode scattering rate (blue dashed line) for electron energy larger than 0.12 eV. Also notice that the interface mode scattering curve shows more than two of the step-like features. This is due to the emission threshold energy split between the symmetric and asymmetric interface modes. With *d* = 5, the wave vector *q* is only large enough to satisfy the emission condition at phonon frequencies of *ω* = *ω*_TO(LO),res_. However, with smaller *d*, this is no longer the case and the condition is satisfied with phonon frequencies slightly away from the resonant frequencies. As the symmetric and asymmetric interface modes are defined in separate phonon frequencies, except at the limit of *ω* → *ω*_TO(LO),res_, the threshold energies are split and causes the scattering rate curve to show more step-like increases.

**Figure 6:**
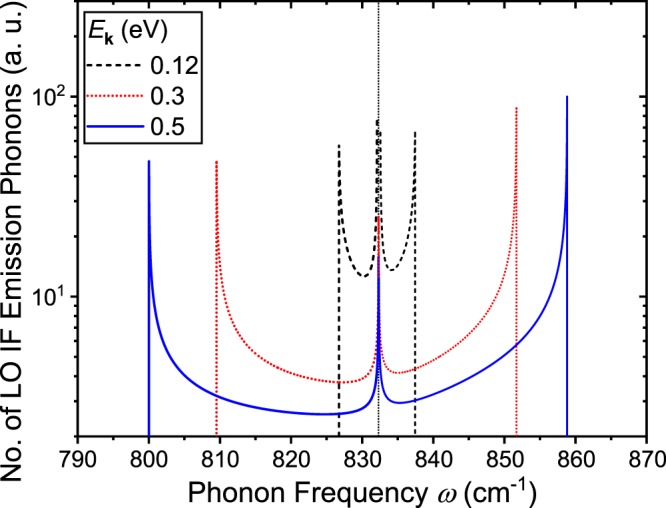
Number of produced interface phonons as a function of phonon frequency for different electron energies *E*_**k**_ = 0.12 (black dashed line), 0.3 (red dotted line), and 0.5 eV (blue solid line) are shown. Only the interface phonons involved in the LO emission phonon process are plotted. The LO phonon resonant frequency is indicated as the vertical dotted line. The *y*-axis represents the integrand of Eq. (9) or equivalently the number of phonons emitted with each phonon frequency.

To further examine the interface phonons that are emitted in the LO emission scattering process, the integrand of Eq. (9) as a function of phonon frequency for different electron energies *E*_**k**_ = 0.12, 0.3, and 0.5 eV is presented in Fig. 6. The plotted curves represent the number of phonons produced with each phonon frequency. The *x*-axis in this figure is shown in the units of cm^−1^, but should be interpreted as s^−1^ such that the integral of the curves over the phonon frequency results in the scattering rates represented in s^−1^.

The vertical dotted line in Fig. 6 indicates the LO resonant frequency *ω*_LO,res_ = 832.3 cm^−1^. Phonons with frequency larger (smaller) than this resonant frequency are symmetric (asymmetric) modes. First, at *E*_**k**_ = 0.12 eV, which is slightly above the LO phonon emission threshold energy *E*_LO,res_ = 103.2 meV, the phonons that are emitted show a small span of frequencies with the ones very close to *ω*_LO,res_ = 832.3 cm^−1^ being the majority. As the electron energy increases, the scattering process starts to produce more phonons with frequencies farther away from the resonant frequency. With *E*_**k**_ = 0.5 eV, a large amount of phonons with *ω*_LO,res_ ~ 832.3 cm^−1^ are still generated but the majority of the phonons possess frequencies that are approximately 30 cm^−1^ away from the resonant frequency. Returning to Fig. 3, we see that the group velocity of these LO interface phonon modes is around 30 km/s which is considerably large compared to the maximum group velocity of confined phonons (7 km/s) but still quite small compared to the maximum group velocity of the available interface phonons (138 km/s). In order to utilize the interface phonons with larger group velocity, the electron energy must be increased. However, with GaN thickness set to *d* = 5 nm, to utilize the entire span of available interface phonons and generate phonons with the maximum group velocity, calculation results show that the electron energy has to increase up to physically unrealistic values as high as *E*_**k**_ = 100 eV. An alternative way to produce interface mode phonons with high group velocity may be increasing the GaN thickness *d* as was suggested from the dispersion relation shown in Fig. 2. The group velocity (in Fig. 3) scales linearly with *d*; for example, if the thickness increases two-fold to *d* = 10 nm, the group velocity at each phonon frequency also increases to twice of the value calculated at *d* = 5 nm.

**Figure 7:**
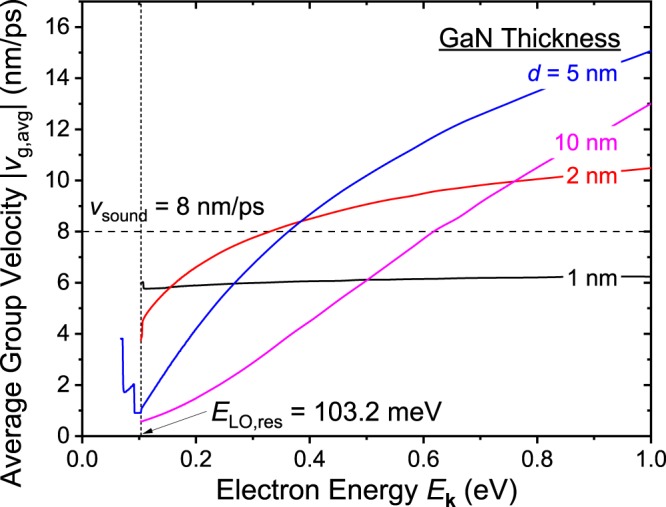
Average group velocity of the emitted interface and confined mode phonons with different GaN thickness d = 1, 2, 5, and 10 nm as a function of electron energy *E*_**k**_ is shown. As a reference, the longitudinal acoustic phonon propagation velocity along the *c*-axis [0001] is indicated as the horizontal dashed line. At low energies right above *E*_LO,res_ = 103.2 meV, heterostructures with thinner GaN thickness *d* shows a larger average group velocity. In contrast, as electron energy increases, it shows that the average group velocity of heterostructures with thicker *d* saturates at a larger value.

The relation between the GaN thickness *d*, phonon dispersion relation, interface phonon mode group velocity, and electron energy *E*_**k**_ must all be taken into account to understand if the nonzero interface mode phonons can be exploited to effectively dissipate heat of the system. To this end, we calculate and present in Fig. 7 the average group velocity of the emitted interface and confined mode phonons with different GaN thicknesses *d* = 1, 2, 5 and 10 nm as a function of electron energy *E*_**k**_. Although a GaN thickness of 10 nm may be difficult to grow with the existing technology due to the strain associated with the lattice mismatch in AlN/GaN/AlN structures, we include it in our analysis to show the trend^23^. The procedure of obtaining the average group velocity is as follows: First, to the integrand of Eq. (9) (shown in Fig. 6), we multiply the phonon frequency dependent group velocity and evaluate the integral to obtain the sum of group velocities of all emitted phonons. After adding up all contributions from each interface and confined, TO and LO, symmetric and asymmetric phonon modes, we divide it with the total scattering rate to obtain the average group velocity. The average group velocity *v*_g,avg_ may be expressed as:

16 $${v}_{g,avg}=-\,\frac{2m{e}_{0}^{2}{\tau }^{e}}{{\hslash }^{2}}\sum _{n}{\int }_{{\omega }_{1}}^{{\omega }_{2}}\frac{[N(\omega )+1]D(q,\omega )\sigma [\{\frac{\omega }{{q}^{2}}-\frac{\hslash }{2m}\}\frac{dq}{d\omega }-\frac{1}{q}]}{[\frac{q}{2}-\frac{m}{\hslash }\{\frac{\omega }{q}-\frac{d\omega }{dq}\}]\sqrt{\frac{1}{m}\chi ({\bf{k}},q,\omega )}}{v}_{g}d\omega $$

where *τ*^e^ is the emission scattering rate obtained from Eq. (9), and *v*_g_ is the group velocity of the corresponding phonon mode. As a reference, the longitudinal acoustic (LA) mode phonon’s sound velocity in GaN along the optical *c*-axis [0001] is shown as the horizontal dashed line^24^. Among all GaN thicknesses considered, we see a general trend of increasing average group velocity of emitted phonons as the electron energy increases. This is due to the increase of number of interface phonons with higher group velocities (i.e., phonons with frequency away from resonant frequencies) that are involved in the electron–phonon scattering process. Although in Fig. 6, we only show the case for *d* = 5 nm, similar trend was observed for all GaN thicknesses: with higher electron energy, more phonons with higher group velocity are emitted.

For the *d* = 5 nm case, at energies smaller than 103.2 meV, a step-like change in average group velocity is shown. This is due to the contribution of different phonon modes with different group velocities. As the electron energy increases, at each emission threshold energy *E*_TO_ = 67.6 meV, *E*_TO,res_ = 71.7 meV, *E*_LO_ = 91.6 meV, and *E*_LO,res_ = 103.2 meV (see Fig. 4), different modes of phonons start to emit, and their contributions appear in the curve as step-like shapes. At energies larger than *E*_LO,res_, most of the phonons that are emitted are confined and interface LO phonons, and the average group velocity is determined by the competition between these two factors. The step-like changes for the curves of different GaN thicknesses are omitted in the figure. For the *d* = 1 nm case, at low electron energies right above *E*_LO,res_ = 103.2 meV, the average group velocity is the largest compared to the velocities with different thicknesses but has already almost saturated to its largest value which is around 6 nm/ps. As the thickness increases, the group velocity starts at a smaller value but increases more steadily and saturates at a larger value (the increase rate of velocity for the *d* = 10 nm case does not even start to saturate at *E*_**k**_ = 1.0 eV suggesting that it has potential to reach a higher value). From the curves, therefore, depending on the range of available electron energies and their distribution in energy, an appropriate GaN thickness can be selected such that the interface phonons can contribute to the heat dissipation process. Putting the electron energies and distribution aside, we see that the average group velocity can easily be engineered to a value comparable to the acoustic phonon’s sound velocity. Considering that the acoustic phonons are the major contributor of thermal conduction in semiconductor materials, the results suggest that the generated optical phonons of these systems may also contribute largely to the thermal conduction.

## Conclusion

In conclusion, we have theoretically studied the interface and confined mode optical phonons of a wurtzite AlN/GaN/AlN double heterostructure and their interaction with electrons based on the uniaxial dielectric continuum model. The phonon dispersion relation of these phonon modes and the electron–phonon scattering rates are calculated numerically to derive the average group velocity of the emitted phonons to explore the possibility of exploiting the interface mode phonons as an additional heat dissipation channel. Our estimations show that the average group velocity of phonons that are emitted through electron–phonon scattering processes with electron energy slightly larger than the threshold energy (*E*_**k**_ ≈ *E*_LO,res_) is very small (for the *d* = 5 nm case, 0.9 nm/ps) compared to the LA phonon propagation velocity of bulk GaN (*v*_sound_ = 8 nm/ps). This is due to the dispersion relation of interface mode phonons which shows curves that converge to the resonant phonon frequency at large phonon wave vector *q*. With larger electron energies, the average optical group velocity can exceed the acoustic phonon velocity starting from *E*_**k**_ = 0.35 eV. Given the energy distribution of electrons, we report that the quantum well thickness can be engineered to exploit the interface mode phonons, which can propagate a distance of few tens of nanometers before decaying into heat-carrying acoustic phonons.
